# Determinants of food insecurity among smallholder farmers in North Central Nigeria: policy implications

**DOI:** 10.3389/fnut.2026.1790322

**Published:** 2026-04-16

**Authors:** Marcus Olaitan Ogunfolaju, Tijani Abdulhamid Ahmed, O. Ebenehi

**Affiliations:** 1 Centre for Inclusive Social Development (CISD), Abuja, Nigeria; 2 Department of Agricultural Economics and Extension, Prince Abubakar Audu University, Ayingba, Nigeria; 3 Department of Agricultural Extension and Rural Development, Federal University, Dutsin Ma, Katsina, Nigeria

**Keywords:** determinants, food security, Household Food Insecurity Access Scale, Nigeria, smallholder farmers

## Abstract

This study investigates the determinants of food insecurity among smallholder farmers in North Central Nigeria. A multi-stage sampling procedure was used to select 812 respondents for the study. The study employed the Household Food Insecurity Access Scale (HFIAS) to measure food security status. An ordered probit regression model was used to analyze the relationship between food security status and key socioeconomic and agricultural factors. The results revealed that the sex of the household head, marital status, level of education, farm size, and years of farming experience significantly influenced food security status. Male-headed, married, more educated, experienced farmers with larger landholdings were more likely to be food secure. In contrast, variables such as household size, land ownership, and income were not statistically significant. Households experienced high levels of food insecurity, such as uncertainty about their food supply, concerns about poor food quality, and experiences of insufficient food intake. The findings underscore the need for policies promoting equitable access to education, land, and extension services, especially for women and young farmers, which are critical to improving household food security in the region.

## 1 Introduction

Global hunger and food insecurity have surged in recent years, dominating international discussions (1–3). Food insecurity refers to the inability to reliably obtain or afford sufficient food (4). Its opposite, food security, was defined as ensuring all people, at all times, have physical and economic access to enough safe, nutritious food to meet dietary needs and preferences for a healthy, active life (5). Household Food Insecurity (HFI) involves uncertain or inadequate food availability, use, or access, often leading to nutrient deficiencies, poor nutrition, reduced individual productivity, weaker labour forces, and diminished human capital (6–8).

A Food and Agriculture Organization (FAO) report highlighted an alarming rise in 2020 under the COVID-19 pandemic, with the prevalence of undernourishment (PoU) climbing 1.5% to affect 9.9% of the world’s population, derailing progress toward Zero Hunger by 2030. Asia bore the heaviest burden (418 million undernourished), followed closely by Africa (282 million). Worldwide, severe food insecurity jumped from 8.3% (604.5 million people) in 2014 to 11.9% (927.6 million) in 2020, with Africa recording the second-highest rates after Asia (5).

In Africa, moderate-to-severe food insecurity affected 25.9% of the population (346.6 million) in 2020, up from 17.7% (203.5 million) in 2014. West Africa saw the sharpest increase, from 8.6% (29.6 million) to 28.8% (115.7 million), while North Africa had the lowest sub-regional rates, rising modestly from 29.7% (65.1 million) to 30.2% (74.5 million) (5).

Africa faces acute challenges, with most countries (except North Africa) scoring moderate (40–59.9/100) to weak (20–39.9/100) on the 2020 Global Food Security Index (GFSI) for 32 of 113 countries assessed. Over 83% of weakly scoring nations were from sub-Saharan Africa (SSA), signaling a regional crisis. North African countries like Morocco (62.0), Algeria (61.8), Tunisia (61.4), and Egypt (61.1) achieved “good” scores (60–79.9), but SSA nations such as Sierra Leone (37.0), Malawi (36.7), Zambia (36.6), and Sudan (36.0) ranked among the lowest globally (9).

Nigeria exemplifies these struggles, driven by macroeconomic woes, conflicts, insecurity, and COVID-19 impacts (1, 3). With a population of about 209.7 million as of October 2021, 41% live in extreme poverty (USD 1.90/day threshold), mostly rural where smallholder farmers predominate. Despite leading global production of cassava, yam, and cowpea, food production gains have not curbed insecurity. Nigeria’s GFSI score dipped slightly from 40.9/100 in 2012 to 40.1 in 2020 (9), ranking 100th worldwide and 22nd in Africa. Its 2020 Global Hunger Index score of 29.2 (serious) reflects undernourishment rising from 9.1% in 2000 to 12.6% in 2020, jeopardizing SDG 2 goals for ending hunger and promoting sustainable agriculture by 2030 (1, 2, 10).

Several studies have probed Household Food Insecurity in Nigeria using micro- and macro-level data, including food insecurity status, food poverty indices, consumption scores, and the Household Food Insecurity Access Scale (HFIAS) (11–16). Owoo (17) mapped spatial patterns via fixed-effects regression, while Obayelu and Oyekola (18) applied HFIAS to urban non-farmers in Ibadan slums. Globally, HFIAS has assessed insecurity in places like Kassala State, Sudan, and Takhar, Afghanistan (19, 20).

This study addresses a gap in North Central Nigeria’s food security research by using primary data from smallholder farming households and the HFIAS to examine determinants of food insecurity, offering targeted policy implications.

This study, therefore, seeks to assess the determinants of food security among smallholder farmers in North Central Nigeria. The specific objectives are to:

(i) Describe the socioeconomic characteristics of the smallholder farmers in the study area.
(ii) Assess the food insecurity access-related domains among farming households in the study area.
(iii) Identify the determinants of food insecurity among farming households in the study area.

## 2 Research methodology

### 2.1 Study area

The study was conducted in North Central Nigeria. North Central Nigeria is made up of six states (Benue, Kogi, Kwara, Nasarawa, Niger and Plateau) and the Federal Capital Territory (FCT), Abuja. The North Central zone of Nigeria lies predominantly within the Guinea savanna ecological region, although its vegetation extends across three savanna belts, namely the Guinea, Sudan, and Sahel savannas (21). This ecological diversity supports the widespread cultivation of both cereal and root crops in the area (22). The region comprises Plateau, Kwara, Benue, Niger, Kogi, and Nasarawa States, as well as the Federal Capital Territory (Abuja). Geographically, it is situated between latitudes 7°00′ and 11°30′ North of the equator and longitudes 4°00′ and 11°00′ East of the Greenwich Meridian. Annual rainfall in the region typically ranges from 1,200 mm to 1,500 mm, while temperatures remain relatively high throughout the year, except during the Harmattan period, which occurs between November and February.

### 2.2 Sampling procedure

A multistage sampling procedure was employed to select 812 smallholder farmers. The total sampling frame was 2,410 registered smallholder farmers across 28 selected cells in the three states. From this, a final sample size of 812 respondents was drawn. Specifically, 318 respondents were selected from Niger State, 237 from Kwara State, and 257 from Kogi State using the sample size formula as shown in Table 1.

**Table 1:** Sampling procedure.

Sampling procedure for selection of respondents	Selected states	50% of zones from each state	20% of blocks from selected zone	20% of cells from selected block	Total number of registered farmers in selected cell (sample frame)	Sample size [*n* = *N*/1 + *N*(*e*^2^)]
	Niger	Zone A	Mokwa	Bokani	80	23
				Mokwa	75	21
			Kacha	Badegi	79	23
				Kacha	80	23
			Lavun	Kutigi	75	21
				Lanle	83	24
		Zone C	Ibbi	Ibbi	81	23
				Zugurma	77	22
			Kontagora	Kontagora	80	23
				Tungan Wawa	79	23
			Wushishi	Wushishi	76	22
				Kodo	78	22
			Zungeru	Zungeru	85	24
				Kaliko	85	24
					*Total = 1,113*	*= 318*
	*Kwara*	Zone B	Lafiaji	Lafiaji	97	41
			Lade	Lade	83	35
		Zone C	Iporin	Oke Oyi	98	41
				Oke Ose	87	37
				Agbeyangi	96	40
			Shao	Sobi	103	43
					*Total = 564*	*= 237*
	*Kogi*	Zone B	Egume	Anyigba	85	30
				Egume	108	38
			Ankpa	Ojapata	80	28
				Ankpa	110	39
		Zone D	Alloma	Ejule	90	32
				Umomi	85	30
			Okpo	Imane	95	33
				Okpo	80	27
					*Total = 733*	*=257*
*Total*	*3*	*6*	*15*	*28*	*2,410*	*812*

## 3 Data collection and measurement of variables

Data were collected through a structured questionnaire. The food security status of the respondents was measured using the Household Food Insecurity Access Scale (HFIAS).

### 3.1 Measuring food access through HFIAS

The Household Food Insecurity Access Scale (HFIAS), developed by the Food and Nutrition Technical Assistance (FANTA) Project and partners (23), evaluates household economic access to food, preferences, supply-related anxiety, and quantity. It features nine occurrence questions that progressively gauge food insecurity severity, each followed by a frequency question assessing how often the condition arose in the past 30 days (rarely: 1–2 times; sometimes: 3–10 times; often: ≥10 times), skipped if the occurrence was denied. These questions apply universally to all household members, regardless of age.

HFIAS has proven effective in distinguishing secure from insecure households across countries. The generic questions capture insecurity experiences, enabling prevalence assessment, population categorization by severity, and tracking changes over time.

### 3.2 Household Food Insecurity Access Prevalence (HFIAP)

HFIAP, a key HFIAS output, classifies households into four distinct levels: food secure (FS), mildly food insecure (MiFI), moderately food insecure (MoFI), and severely food insecure (SFI). Households progress to higher insecurity as they affirm more severe conditions or experience them more frequently within 30 days. Coding assigns 0 to frequency responses for denied occurrences (e.g., if Q1 = 0, then Q1a = 0) before grouping. While total HFIAS scores (continuous) summarize experiences, only HFIAP assigns categorical status based on the most severe responses.

#### 3.2.1 HFIAP categories

*Food Secure (FS)*: No insecurity or only rare worry about food (Q1 = 0 or Q1 = 1 with Q1a = 1).

*Mildly Food Insecure (MiFI)*: Occasional/frequent worry (Q1 = 1, Q1a = 2–3); and/or unattractive/preferred foods unavailable (Q2 = 1, any Q2a); limited variety rarely/sometimes (Q3 = 1, Q3a = 1); or undesirable foods rarely (Q4 = 1, Q4a = 1).

*Moderately Food Insecure (MoFI)*: Undesirable foods or limited variety sometimes/often (Q3 = 1, Q3a = 2–3; Q4 = 1, Q4a = 2–3); and/or fewer/smaller meals rarely/sometimes (Q5 = 1, Q5a = 1–2; Q6 = 1, Q6a = 1–2); but no severe conditions (Q7–9 denied).

*Severely Food Insecure (SFI)*: Frequent meal reductions (Q5 = 1, Q5a = 3; Q6 = 1, Q6a = 3); and/or any extreme conditions, no food, bedtime hunger, or full-day hunger—even rarely (Q7–9 affirmed, any frequency).

### 3.3 HFIAS domains and items

The nine items are grouped into three experiential domains, progressing from milder to severe manifestations of food insecurity:

*Anxiety and Uncertainty* (3 items): Worry that the household would run out of food (item 1), worry that food would run out before getting money to buy more (item 2), and inability to eat the preferred foods due to lack of resources (item 3).

*Insufficient Food Quality* (3 items): Eating a limited variety of foods due to lack of resources (item 4), eating foods they did not want to eat (item 5), and eating unappealing or undesirable foods (item 6).

*Insufficient Food Intake and Severe Hunger* (3 items): Eating smaller meal sizes than needed (item 7), reducing the number of meals eaten in a day (item 8), and going whole days without eating (item 9).

#### 3.3.1 Model specification

The ordered probit model was selected for analyzing determinants of Household Food Insecurity, as the dependent variable ( Y ), Household Food Insecurity status is ordinal with four progressively severe categories: 1 = food secure, 2 = mildly food insecure, 3 = moderately food insecure, and 4 = severely food insecure.

The latent (unobserved) propensity toward food insecurity for household *i* is modelled as:


$$\begin{array}{l} Y\_i*=\beta 0+\beta 1\mathrm{Age}\_i+\beta 2\mathrm{Sex}\_i+\beta 3\text{MaritalStatus}\_i\\ +\beta 4\text{HHSize}\_i+\beta 5\text{Education}\_i+\beta 6\text{FarmSize}\_i\\ +\beta 7\text{FarmExperience}\_i+\beta 8\text{Income}\_i+\varepsilon \_i\;\; \end{array}$$ (1)

Where:

- Y_i* = latent food insecurity index
- *βⱼ* = parameters to estimate (*j* = 0 to 8)
- *ε_i* ~ *N*(0,1) = error term (standard normal)

$$\begin{array}{l} \text{The observed outcome}\;Y\_i\;\text{maps from the latent}\;\\ \text{variable}\;\mathrm{via}\;\text{estimated}\;\mathrm{cut}-\text{points}: \end{array}$$

Y_i=1ifY_i∗≤μ₁(Food Secure)

=2ifμ₁<Y_i∗≤μ₂(Mildly Food Insecure)

=3ifμ₂<Y_i∗≤μ₃(Moderately Food Insecure)

=4ifY_i∗>μ₃(Severely Food Insecure) (2)

Probability of category *j* is:


$$\begin{array}{l} P(Y\_i=j)=\Phi (\mathrm{\mu j}-X\_\mathrm{i\beta})\\ -\Phi (\mathrm{\mu j}-1-X\_\mathrm{i\beta}),j=1,2,3,4\;\; \end{array}$$ (3)

WhereΦ(·)=standard normalCDF,μ0=−∞,μ4=+∞.

## 4 Results and discussion

### 4.1 Socioeconomic characteristics of respondents

#### 4.1.1 Farm size

Table 2 shows that the majority (61.1%) of the respondents cultivated less than 1 ha of land. The average land area cultivated by the respondents was 1.6 ha. This study supports the findings that the majority of farmers in North Central Nigeria have a farm size of less than 2 ha (24). Smallholders often face difficulties achieving self-sufficiency, especially when yields are low or markets are inaccessible. Farm size significantly affects output volume and, by extension, household food availability and income levels (8). Households with larger farms typically enjoy greater resilience and surplus for sale, which strengthens both food and financial security.

**Table 2:** Socioeconomic characteristics.

Variables	Percentage	Mean (Std Dev.)
Farm size (ha)		1.6 (1.71)
Less than 1.0	61.1	
1.1–2.0	15.3	
2.1–3.0	5.5	
3.1–4.0	2.3	
4.1–5.0	15.8	
Min. = 0.1; Max. = 5.0		
Monthly income (Naira)		203,817.7 (167,857.08)
5,000–50,000	27.9	
51,000–150,000	19.1	
151,000–300,000	15.6	
301,000–500,000	34.6	
501,000–600,000	3.1	
Experience in farming (years)		17.4 (10.08)
1–10	33.3	
11–20	38.4	
21–30	18.0	
Above 30	10.3	
Land ownership for farming		
Yes	82.6	
No	17.4	

Source: Field Survey, 2024.

### 4.2 Income

Table 2 indicates that respondents earned a mean monthly income of 203,817.71 Naira. The estimated average daily income was N6,793.92. This is equivalent to $4.37 per day considering CBN exchange rate as at the time of the analysis. This is higher than the World Bank International poverty line of US $1.9 a day reported for Sub-Saharan African countries.

Income level is a critical determinant of food security, especially in market-reliant food systems where household access depends more on purchasing power than subsistence production (25). Moreover, low-income farmers often depend on less nutritious, cheaper food and are unable to adequately invest in farm productivity, further perpetuating the cycle of poverty and food insecurity (26).

#### 4.2.1 Land ownership

Table 2 reveals that most (82.6%) of the respondents own the land that they use for their farming activities. Ownership of land can improve land use and management. This corroborates the findings of Mdoda and Gidi (27) that the majority of farmers own land and land ownership is one of the crucial ways towards sustainable development for productivity-enhancing investment, land management, and eventually improving access to credit markets and financial institutions through using of land as security. Land ownership provides security and incentivizes farmers to invest in long-term productivity-enhancing practices such as soil improvement, irrigation, or agroforestry (5).

### 4.3 Food insecurity access-related domains

**Figure 1:**
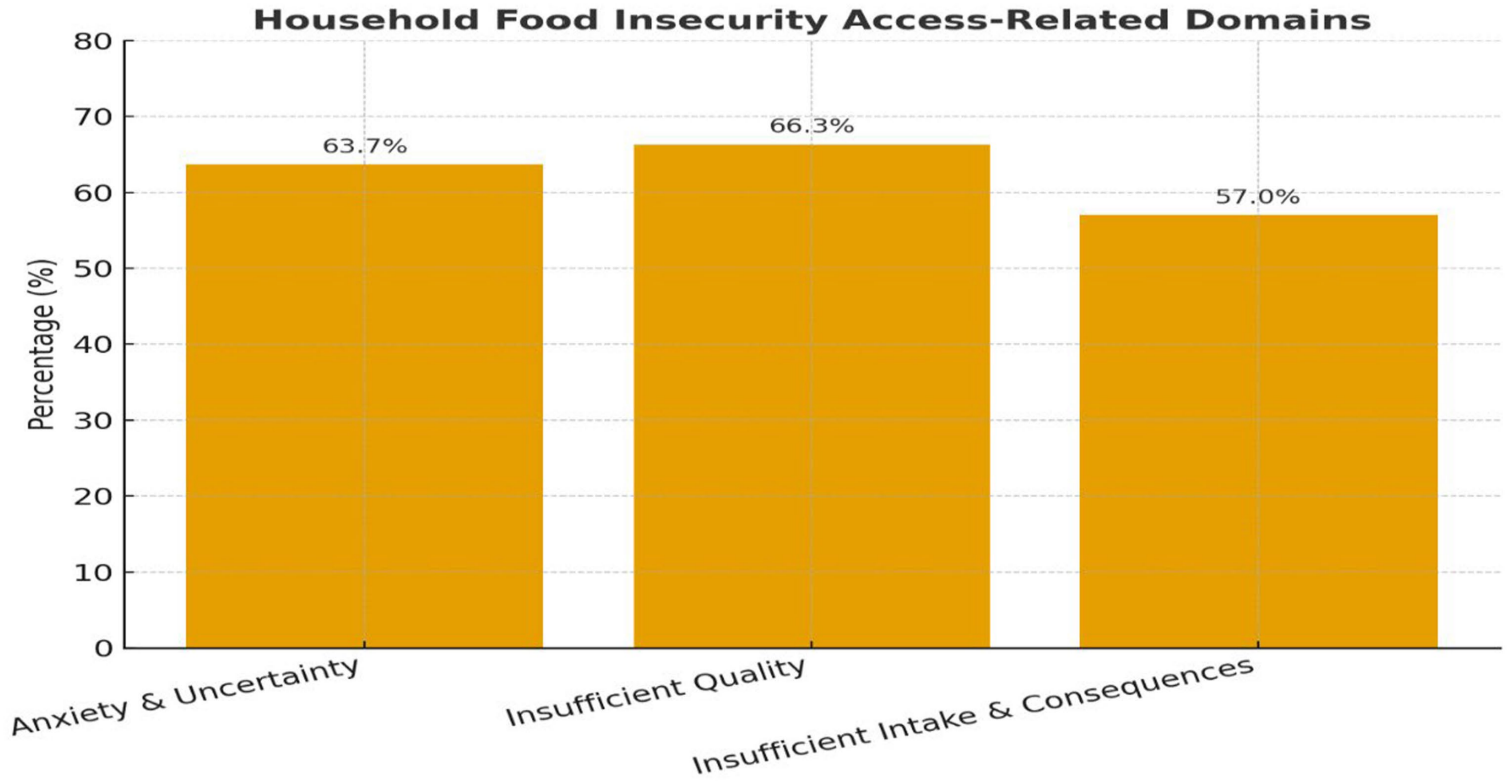
Food insecurity access-related domains.

The findings in Figure 1 reveal high levels of Household Food Insecurity across all three areas of the food insecurity access-related domains. The majority reported feelings of uncertainty and anxiety about their food supply (63.7%), concerns about poor food quality (66.3%), and experiences of insufficient food intake with physical effects (57.0%). These results highlight the multidimensional aspects of food insecurity, showing that households are not only dealing with the psychological stress of potential shortages but are also facing declines in both dietary quality and quantity. Anxiety often emerges before severe reductions in food intake, reflecting a coping stage where households anticipate constraints and attempt to mitigate risk (28, 29). The persistence of food-related worry in the present study suggests chronic instability in food access, which may also have mental health implications for household members.

The majority of respondents (66.3%) reporting insufficient food quality reveals that many households are forced to compromise on dietary diversity and accept less-preferred foods. This mirrors global and regional evidence that quality is typically sacrificed before quantity during periods of constrained access (30). Such compromises can lead to micronutrient deficiencies and diet-related health challenges, even in households where caloric sufficiency is only moderately affected (3). Given the established links between dietary diversity and nutrition outcomes, the loss of food quality poses a serious public health concern.

### 4.4 Determinants and food security status

The result in Table 3 shows that the overall model was statistically significant with a likelihood ratio chi-square of 179.36, indicating that the model fits the data better than a model with no predictors. The *R*^2^ value of 0.1233 suggests a modest explanatory power, which is typical for cross-sectional models involving complex social phenomena like food insecurity.

**Table 3:** Determinant and food security status.

Variable	Coef. (*β*)	Std. Error	*t*-value
Age	−0.0088944	0.0053342	−1.67*
Sex	0.3860468	0.1126513	3.43*
Marital status	−0.5308313	0.1318923	−4.02*
Household size	−0.0065611	0.0150226	0.44
Education	−0.0426018	0.0093756	−4.54*
Land ownership	0.0563972	0.1228651	0.46
Farm size	−0.1678289	0.0308976	−5.43*
Farming experience	−0.0173107	0.0059014	−2.93*
Income	−3.77e−07	3.04e−07	−1.24
/cut1	−1.913262	0.3438736	
/cut 2	−1.622784	0.3425702	

Model summary.

Number of observations = 812.

LR chi^2^(9) = 179.36.

Prob > chi^2^ = 0.0000.

Pseudo *R*^2^ = 0.1233.

Log likelihood = −637.56984.

Significant at ^*^p ≤ 0.01, ^**^p ≤ 0.05, ^***^p < 0.10.

Among the independent variables, sex of the household head emerged as a statistically significant determinant (*β* = 0.386). The positive coefficient implies that being male is associated with a higher likelihood of being food secure (i.e., less likely to fall into more severe categories of food insecurity). This corroborates the findings of Ayogu et al. (31) that male-headed households tended to exhibit higher levels of food security compared to female-headed households.

Marital status also showed a significant negative relationship (*β* = −0.530), suggesting that married individuals were more likely to be food secure compared to those who were single, divorced, or widowed. This aligns with the findings that marital unions improve economic cooperation and labour pooling, which are crucial for sustaining household-level food availability (25).

Years of education (*β* = −0.042) were significant, with a negative coefficient indicating that more years of education reduce the likelihood of food insecurity. Educated farmers are more likely to adopt improved farming practices, engage in income diversification, and access critical agricultural services and information, thereby improving household food access and dietary diversity (26, 32).

The coefficient for farm size was also significant. The negative relationship indicates that farmers with larger landholdings were less likely to experience food insecurity. The positive relationship between farm size and food security may be explained through economies of scale and improved land productivity. Larger farm sizes allow farmers to diversify crop production, reduce risk exposure, and increase total output, thereby improving household food availability and income stability. Farmers with larger landholdings are more likely to adopt improved technologies such as mechanization, irrigation, and improved seed varieties, which further enhance productivity. Larger farms also enable better labor allocation and reduce production costs per unit of output, contributing to improved efficiency and resilience against agricultural shocks This result confirms earlier findings that land size positively affects household food availability and income generation, especially in rural farming contexts where subsistence and market production are intertwined (27, 33). Furthermore, years of farming experience were found to be a significant negative predictor of food insecurity (*β* = −0.017), suggesting that more experienced farmers are less likely to experience food shortages. This may be due to improved agronomic knowledge, resilience to shocks, and accumulated social capital over time (34).

Household size, land ownership, and income were not statistically significant in the model. Household size may not directly influence food security because larger families often balance higher food needs with greater labour availability or multiple income sources, which can offset consumption pressure. Land ownership may not significantly influence outcomes because possession of land does not necessarily ensure productive utilization. Likewise, income may show no significant effect if variations across households are minimal or if food access and welfare depend more on subsistence production and informal support networks than on cash earnings.

## 5 Conclusion

This study examined the determinants of food insecurity among smallholder farmers in North Central Nigeria and revealed that food security outcomes are shaped by a multidimensional set of socioeconomic and structural factors. The findings demonstrate that food security is not solely dependent on agricultural productivity but rather emerges from the intersection of human capital and physical assets. Education and farming experience significantly enhance farmers’ capacity to make informed production and consumption decisions, while farm size provides the physical foundation for improved output and income stability.

Furthermore, the statistical significance of gender and marital status underscores the importance of household labour dynamics and resource pooling in mitigating food insecurity. Male-headed and married households appear better positioned to mobilise labour, diversify livelihoods, and absorb production shocks, thereby reducing vulnerability to food shortages. These findings align with the Sustainable Livelihoods Framework, which emphasizes the role of human, physical, and social capital in determining household resilience and well-being.

The results suggest that food insecurity among smallholder farmers is not uniform but varies across demographic and structural conditions. Female-headed households and farmers with limited experience face greater vulnerability due to constrained access to productive resources, labour shortages, and limited social safety nets.

### 5.1 Recommendations

The findings highlight the need for targeted and inclusive policy interventions to address food insecurity among smallholder farmers in North Central Nigeria. First, government agencies, particularly the Federal Ministry of Agriculture and Food Security, State Ministries of Agriculture, and agricultural development programmes (ADPs), should promote access to productive resources such as land, credit facilities, improved inputs, and extension services for female-headed households and less-experienced farmers who face structural constraints.

Second, agricultural extension agencies, research institutions, and development partners should strengthen farmer education and capacity-building programs to enhance technical knowledge, promote climate-smart agricultural practices, and improve productivity among smallholder farmers.

Third, land administration authorities, local governments, and farmer cooperatives should facilitate improved access to farmland and encourage cooperative farming arrangements to enable smallholder farmers to benefit from economies of scale and improved land productivity.

Finally, policymakers, development partners, and social protection agencies should integrate agricultural support with targeted social protection measures, including input subsidies and livelihood support programs, to strengthen household resilience and improve sustainable food security outcomes.

## Data Availability

The raw data supporting the conclusions of this article will be made available by the authors, without undue reservation.
